# Trace metal contamination in sediment in the Mhlathuze Estuary, northern KwaZulu-Natal, South Africa: effects on the macrobenthic community

**DOI:** 10.1007/s10661-020-08352-9

**Published:** 2020-05-29

**Authors:** Joshua Idowu Izegaegbe, Leon Vivier, Hendrick Mduduzi Mzimela

**Affiliations:** 1 Department of Zoology, University of Zululand, KwaDlangezwa, South Africa; 2 Department of Zoology, Ambrose Alli University, Ekpoma, Nigeria

**Keywords:** Indices, Pollution, Guidelines, Enrichment, Toxicity, M-AMBI

## Abstract

Mhlathuze Estuary constitutes one of the ecological most important estuaries in southern Africa and is regarded as an estuary of high conservation importance. The ongoing expansion of the adjacent industrialized Richards Bay Harbour increases the risk of metal pollution to the estuary. This study provides insight into the extent and sources of trace metal contamination using pollution indices and sediment quality guidelines and the effect on macrobenthic habitat quality. Sediment samples for sediment metal and macrobenthic analysis were collected quarterly during 2016–2017 at five sites in the estuary using a marine-grade Zabalocki grab. Metal concentrations were determined using an ICP-OES. Sediment metal concentrations were consistently highest in the subtidal mudflats and lowest in marine sand at the mouth of the estuary. Concentrations of all metals displayed significant differences between sites (*P* < 0.05). Pollution indices indicated moderate enrichment of Cr at all sites, although the mean pollution load index showed the estuary to be unpolluted. Comparison with sediment quality guidelines revealed that concentrations of Ni and Cr were potentially toxic to biota. Using multivariate analysis, metal concentrations appeared not to significantly affect the macrobenthic community. The multi-metric biotic index M-AMBI proved to be a robust tool in the habitat quality assessment of the estuary. The continuing use of M-AMBI as a biomonitoring tool for ecological management of the estuary is advocated.

**Electronic supplementary material:**

The online version of this article (10.1007/s10661-020-08352-9) contains supplementary material, which is available to authorized users.

## Introduction

Metals are introduced into estuarine and coastal environments largely due to industrialization and economic development (Feng et al. 2002), and they constitute, amongst others, environmental contaminants in aquatic ecosystems. Metals that are of environmental significance as pollution indicators are usually enriched over natural background levels in the water column and in the sediment of estuaries (Biney et al. 1994). The distinction between the sources of trace metal contamination in many coastal environments poses a challenge to coastal managers due to the availability of metals from both anthropogenic and natural sources (Takarina 2011). This led to the introduction of pollution indices based on geochemical normalization (e.g., geoaccumulation index, enrichment factor, and pollution load index) to ecologically evaluate sources and contamination status of pollutants in coastal sediments (Muller 1979; Tomlinson et al. 1980). These indices have proved viable in identifying pollutants of toxicological concern in the marine, estuarine, and freshwater environment (Çevik et al. 2009; Varol 2011; Nowrouzi & Pourkhabbaz 2014; El Nemr et al. 2016; Wei et al. 2016; Sundararajan et al. 2017). In addition to these, sediment quality guidelines have been employed in classifying metal toxicity and as potential signals of pollution effect to resident biota in aquatic ecosystems (Ho et al. 2012; Veerasingam et al. 2012; Xu et al. 2014).

Benthic faunal communities are readily used as bioindicators of sediment metal pollution largely due to their sedentary lifestyle and close intimacy with sediments (Borja et al. 2000; Ryu et al. 2011). Macrobenthos offers many benefits in detecting anthropogenic disturbance in estuarine systems because it can integrate changes in habitat and sediment quality over time (Belan 2004). Thus, macrobenthos can be used to detect the acute and chronic effects of pollution on faunal communities (Dean 2008).

The Mhlathuze Estuary, an estuarine embayment on the subtropical east coast of South Africa, is considered an estuary of national conservation importance due to its size, habitat diversity, biodiversity, and nursery function for many marine organisms (Whitfield 1992; Turpie et al. 2002). Mhlathuze Estuary, formed by division of the original Richards Bay Estuary during harbour construction in 1976, is a proclaimed nature reserve, generally known as the Richards Bay Sanctuary. The northern part of the original estuary was transformed into Richards Bay Harbour, the largest and busiest port in South Africa (Vermeulen & Wepener 1999; Izegaegbe 2020). The Mhlathuze Estuary was ranked 10th out of 250 estuaries in South Africa in the hierarchy of conservation importance due to its size and biodiversity (Turpie et al. 2002). Given the ecological importance of the system, it is alarming that little is known about the recent ecological and pollution status of the system, and no comprehensive assessment to date has been done on macrobenthic community structure as an ecological bioindicator in the system. This is despite the close proximity of the estuary to the vast industrial and shipping complex in Richards Bay Harbour. Ecological studies stressed the importance and significance of habitat diversity and biodiversity of the Mhlathuze Estuary, highlighting the need for the evaluation of the extent of metal pollution in this system (Cyrus, 2001).

Previous studies provided some historical context on the spatial and temporal variations in metal concentrations in water, sediment, and in fish tissues (mullet) (Mzimela et al. 2003; Mzimela et al. 2014). However, very little is known about the present extent and dynamics of sediment metal contamination in the Mhlathuze Estuary. There are concerns that increasing industrial and agricultural activities in the catchment and contamination from the adjacent harbour will threaten the ecological status of the estuary. This is also of particular significance given the proposed large-scale expansion of the harbour over the next two decades, for which extensive dredging will be required. Earlier dredging for expansion of the coal terminal in the harbour resulted in significant sediment intrusion into the Mhlathuze Estuary, resulting in increased turbidities, fine sediment deposition, and loss of *Zostera capensis* stands (Cyrus et al. 2008). Dredging of polluted sediments has been shown worldwide to have toxicological effects due to the resuspension of adsorbed metals, which could lead to loss of biodiversity in the estuary (Cyrus et al. 2008). Ecological quality assessments of estuarine systems such as the Mhlathuze Estuary are vital for decision-making and for establishing remedial measures (Feebarani et al. 2016), given the susceptibility of the estuary to the potential threat of metal contamination from adjacent harbour areas and catchment-related practices.

The objectives of this study were therefore as follows: firstly, to evaluate the sediment metal concentrations in Mhlathuze Estuary, using the available sediment quality guidelines; secondly, to assess the ecological significance of the measured metal concentrations in the estuary using three pollution indices, i.e., enrichment factor (EF), geoaccumulation index (Igeo), and pollution load index (PLI), as indicators of metal accumulation in the system; thirdly, to assess the influence of environmental variables and sediment metal concentrations on the macrobenthic community structure of the Mhlathuze Estuary using multivariate analysis; and lastly, to assess macrobenthic habitat quality using the multi-metric biotic index M-AMBI (Borja et al. 2000).

## Materials and methods

### Study area

**Fig. 1:**
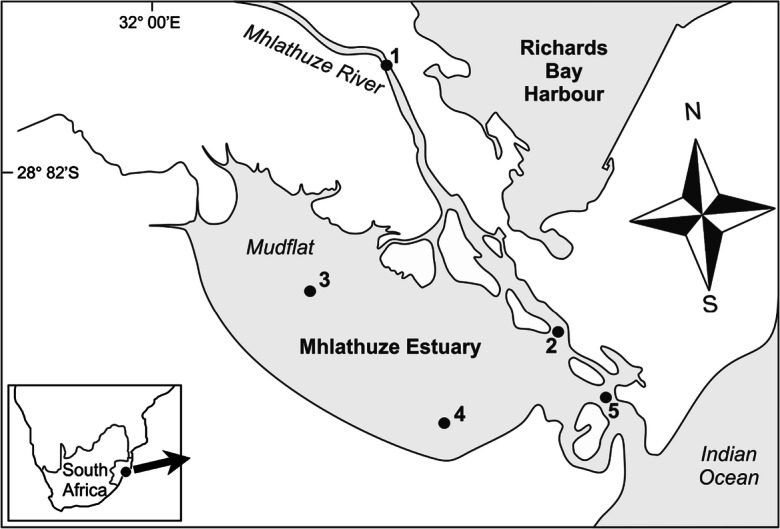
Map of Mhlathuze Estuary on the KwaZulu-Natal coastline, South Africa, indicating the sampling sites during 2016–2017

The 16.9 km^-2^ Mhlathuze Estuary (28^o^ 51′ S; 32^o^ 03′ E) includes 6.5 km^-2^ of mangroves, the largest stand of mangroves in any estuary in South Africa (Naidoo 2016). The large and shallow embayment comprises 75% (5.8 km^-2^) of the intertidal and subtidal habitat in the system (Fig. 1). The mangrove-lined Mhlathuze River enters the estuary from the northwest. Although the estuary is not subjected to direct industrial effluents, activities in the catchment include ongoing dune-mining and extensive sugar-cane, fruit, and forestry agriculture. Rural settlements with poor sanitation along the catchment further increase the potential for metal contamination in the estuary. Sediment transport into the system is reduced due to impoundment, which includes Lake Phobane and the Mhlathuze abstraction weir. Five sampling sites were selected to represent different habitat types in the estuary (Fig. 1). The Mhlathuze River site (site 1) in the upper estuary represented a river-dominated habitat approximately 4 km from the mouth of the estuary. Site 2 is a mangrove-fringed habitat at the junction of the Mhlathuze River and the intertidal embayment, site 3 represented the shallow subtidal mudflat in the embayment, site 4 was the channel habitat off the mudflats, while site 5 represented the sandy habitat at the mouth. It should be noted that even though the estuary is a nature reserve, uncontrolled, and illegal fishing using monofilament gillnets and small-mesh seine nets was observed throughout the study period.

### Sample collection

Sampling for macrobenthos and sediment was done quarterly during 2016–2017 at 5 sites in Mhlathuze Estuary (Fig. 1). Sediment samples for macrobenthos and sediment metal concentrations and granulometry were obtained using a marine-grade Zabalocki type Eckman grab (samples an area of 0.0236 m^2^). Duplicate sediment samples for metal analysis were placed into acid pre-washed honey jars, kept in a cooler at 4 °C, transported to the laboratory, and frozen until further analysis. Five replicate macrobenthic samples were emptied into a 25 l bucket, stirred, and then sieved through a 0.5-mm-mesh sieve. Samples were preserved in 10% formalin, with Phloxine B dye added to facilitate sorting in the laboratory. Sorting of macrobenthic samples was done in the laboratory and where possible, animals were identified to species level using appropriate taxonomic keys. Abundances were converted to densities as no.m^-2^. Water physicochemical parameters including salinity, temperature, pH, dissolved oxygen, and depth were also measured during sampling using a YSI ProDSS multiprobe.

### Sediment analysis

Approximately 0.5 g dry weight (dw) equivalent of duplicate sediment samples was weighed and placed in digestion Teflon vessels before being digested with 10 mL of nitric acid in an advanced microwave (Milestone Ethos Easy Microwave digestion system). Acidified sediment extracts were filtered through a Whatman 47-μm filter paper, diluted to 50 ml with ultra-pure water in 100 ml polypropylene bottles prior to metal analysis. Trace metal concentrations were determined using an ICP-OES. Sediment granulometry was determined using laser diffraction. The total organic carbon (TOC) content (%) of dried and weighed sediment samples was determined by incineration for 6 h at 600 °C.

### Quality control

During sediment digestion, each digestion batch of seven Teflon vessels included a laboratory blank (negative control: only with reagents) and a reference sediment sample (positive control: certified reference material of estuarine sediment BCR 667), to check for the accuracy of the analysis of the sediment samples. Measured and certified concentration was in good agreement. Recoveries ranging from 66.5–89.0% were obtained for the measured trace metals (Cd = 71%, Co = 66.5%, Cr = 70.7%, Cu = 87.2%, Fe = 71.5%, Mn = 89%, Ni = 72%, and Zn = 81%). During metal analysis, calibration verification standards were regularly used to evaluate the calibration curve. The minimum correlation coefficient of the calibration curve accepted was 0.999.

### Data analysis

Following normality (Shapiro-Wilk) testing, one-way analysis of variance (ANOVA) was used to test for differences in mean variables between sites and seasons, followed by Tukey (HSD) post hoc tests. Whenever the assumptions for ANOVA were not fulfilled, a non-parametric Kruskal-Wallis ANOVA by ranks was used. Pearson correlation coefficient was used to measure the linear correlation between variables. Principal component analysis (PCA) (Canoco 5) was used to explore the relationship between sediment metal concentrations and environmental variables. All abiotic data were normalized prior to the analysis. SPSS (IBM, Inc. Chicago, IL) and GraphPad Prism (vs 6.0) were used in data analysis and creating graphs.

Three pollution indices were used to better understand whether the measured concentration of trace metals was from anthropogenic or natural origin. To calculate the enrichment factor (EF), a normalization procedure was performed using iron to compensate for granulometric and mineralogical effects on metal load. This method considers that natural sedimentary loads differ depending on grain size distribution, thus differentiating between natural and anthropogenic input (Ho et al. 2012). The following equation was used to estimate the enrichment of metals from each site using Fe as a normalizer.

$$ EF=\frac{{\left( Metal/ Fe\right)}_{sample}}{Metal/ Fe\Big){}_{background}} $$

where EF = enrichment factor, (Me/Fe) _sample_, and (Me/Fe) _background_ are the metal concentrations (μg/g dw) in relation to Fe levels (μg/g dw) in sediment and background Fe levels, respectively. The background concentration was adopted from Wedepohl (1995), representing the concentration of the metals in the upper continental crust (Rumisha et al. 2012). A deviation from the average crustal values was exempted for Cd only. The background concentration for Cd as provided by Wedepohl (1995) was considered inappropriate given the relatively anomalous enrichment data recorded. It was argued that where a poor relationship between a metal (such as cadmium, arsenic, and mercury) and the normalizer was observed, the highest concentration of the metal measured in sediment from a baseline location can be used to evaluate the concentration above which anthropogenic enrichment is inferred (Newman & Watling 2007; Vivier 2010). Therefore, in overcoming this challenge, the prescribed background value of Cd for the Eastern Cape ecoregion was adopted given that there are no background values for the KwaZulu-Natal ecoregion. Enrichment was assessed using five EF categories (Table 1) (Yongming et al. 2006).

**Table 1:** Contamination categories based on enrichment factor and geoaccumulation index values

Enrichment factor		Geoaccumulation index	
Value	Category	Value	Category
< 1	Background concentration	< 0	Uncontaminated
1–2	Minimal enrichment	0–1	Uncontaminated to moderately
2–5	Moderate enrichment	1–2	Moderately contaminated
5–20	Significant enrichment	2–3	Moderate to heavily contaminated
20–40	Very high enrichment	3–4	Heavily contaminated
> 40	Extremely high enrichment	4–5	Heavily to extremely contaminated
		5–6	Extremely contaminated

The second and third pollution indices (pollution load index and geoaccumulation index) were also calculated using the upper continental crust values with the exception of Cd (as explained above). The pollution load index (PLI) was calculated as the *n*th root of the product of all contamination factors for *n* metals per sample. Contamination factor (CF) is the ratio of the concentration (C) of each heavy metal per site over the concentration of the associated background level (Hakanson 1980).

where CF = Cmetal

                   C_background_

Therefore, PLI is calculated as:

$$\mathrm{PLI}=\left(\mathrm{CF}1\times \mathrm{CF}2\times \mathrm{CF}3\dots \dots \dots \dots \mathrm{CF}\mathrm{n}\right)\ 1/n$$

where *n* is the number of metals and CFI, CF2…… are contamination factors of the various metals in a sample (Tomlinson et al. 1980).

For ecological relevance, PLI was interpreted as follows: PLI < 1 means no pollution, PLI of 1–2 means moderate pollution, PLI of 2–3 means heavy pollution, and PLI > 3 means extremely heavy pollution (Liu et al. 2013).

The geoaccumulation index (Igeo) was used to quantify trace metal accumulation in sediment.

$$Igeo=\frac{Log_2(Cn)}{1.5\times Bn}$$

where Cn = concentration of the metal, Bn = geochemical background value, and the factor of 1.5 accounts for possible variation caused by lithogenic effects. The various classes of Igeo are presented in Table 1.

The concentration of trace metals in sediments was also compared with sediment quality guidelines (SQGs) for Australia, New Zealand, and USA (Burton Jr, 2002) for subtropical systems to ascertain if the sediment metal concentrations exceed acceptable levels and are potentially toxic to aquatic life. International guidelines were adopted because there are no official SQGs for South Africa. The threshold effect level (TEL) and effects range low (ERL) are two critical values employed to evaluate the risk posed to aquatic life by trace metals in sediments. TEL values represent chemical concentrations below which adverse effects are rarely observed (Macdonald et al. 1996), while ERL represents the concentration at which toxicity may begin to be observed in sensitive organisms (Long et al. 1995). To assess the relative toxicity of metals based on the critical values, metal concentrations at each site were divided by the TEL and ERL values to derive toxic unit values (TC). A TC value greater than one indicates that the sediments are potentially toxic to marine fauna (Rumisha et al. 2012).

The macrobenthic community structure in the Mhlathuze Estuary was evaluated in relation to sediment metal concentrations across the five sites. For a detailed description of the macrobenthic community structure of the Mhlathuze Estuary, see Izegaegbe et al. (2020). Canonical Correspondence Analysis (CCA) (Canoco 5) was used to evaluate the correlation between sediment metal concentrations, environmental variables, and the macrobenthic community pattern. The Multivariate AZTI Marine Biotic Index (M-AMBI) was used to assess the macrobenthic habitat quality of the different sampling areas in the Mhlathuze Estuary. The M-AMBI, a derivative of the AZTI Marine Biotic Index (AMBI) (Borja et al. 2000), is widely used as an effective biomonitoring tool in evaluating the ecological status of coastal environments (Borja et al. 2000). These indices are important components in the European Water Framework Directive for the protection and management of estuarine and coastal waters (Kennedy et al. 2011) and in categorizing the extent of habitat degradation (Sigamani et al. 2015). M-AMBI incorporates species diversity (Shannon-Wiener diversity), species richness, and the relative sensitivity of the macrobenthic taxa present in each sample to habitat disturbance and pollution into a multi-metric index to assess macrobenthic habitat quality (Muxika et al. 2007; Li et al. 2013). For interpretation of the results, the following M-AMBI categories of habitat quality were used: high (> 0.77), good (0.53–0.77), moderate (0.38–0.53), poor (0.20–0.38), and very poor (< 0.2) (Borja & Tunberg 2011).

## Results

### Water and sediment characteristics

**Fig. 2:**
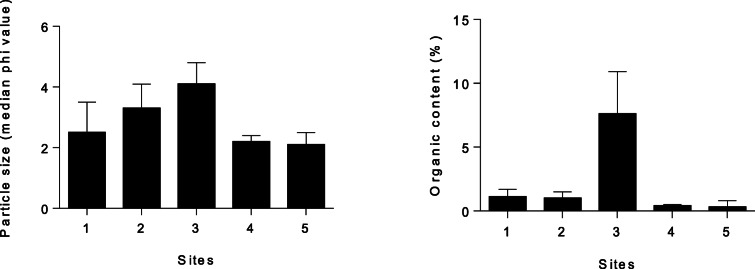
Mean (± 1STD) particle size (median phi value) and organic content (%) of sediments at five sampling sites in Mhlathuze Estuary

Salinities recorded during the study period reflected the strong marine influence in the system, ranging between 26.1 and 35.5, with the highest salinity values predictably at the mouth (site 5) and the lowest in the upper reaches (site 1). There were no significant differences in mean salinity per site (*F* = 2.2, *P* = 0.087), indicating relatively stable salinities in the system due in part to the permanently open mouth and also due to limited freshwater inflow past the abstraction weir. The subtidal embayment (site 3) was characterized by muddy sediment (median phi > 4), while site 2 was very fine sand and sites 1, 4, and 5 were fine sand (Fig. 2). There were significant differences in mean sediment grain size (median phi) between sites (*F* = 11.4, *P* < 0.001). Organic content (TOC) was significantly higher at site 3 (> 5%) compared to the other four sites (*F* = 33.0, *P* < 0.001), reflecting the consolidated muddy substrate of the subtidal mudflats (Fig. 2).

### Sediment metal concentrations

Mean concentrations of trace metals in sediment are presented in Table 2. Concentrations of Co, Pb, Ni, Al, Cu, Fe, Mn, and Zn were consistently highest at site 3 and lowest at site 5. The highest Cr concentrations were recorded at site 2. Significant differences were observed in mean sediment metal concentrations between sites for all metals (Table 2). Analysis of variance (ANOVA) showed particularly high *F* values for Cu, Co, Cd, Cr, Ni, and Zn (Table 2). There were however no significant differences between seasons in the mean of any of the studied metals (*P* > 0.05).

**Table 2:** Mean (± 1STD, *n* = 80) concentrations (μg/g) of sediment metals at five sites in the Mhlathuze Estuary. Analysis of variance (*F* value) is also included

Sites	Co (μg/g)	Pb (μg/g)	Cd (μg/g)	Cr (μg/g)	Ni (μg/g)	Al (μg/g)	Cu (μg/g)	Fe (μg/g)	Mn (μg/g)	Zn (μg/g)
1	5.6^a^ (± 1.8)	12.4^a^ (± 4.4)	0.2^a^ (± 0.1)	41.3^a^ (± 12.9)	12.6^a^ (± 3.6)	12961^a^ (± 5041)	10.1^a^ (± 3.8)	14619^a^ (± 7600)	328^a^ (± 219)	14.9^a^ (± 4.5)
2	8.1^b^ (± 2.1)	17^a^ (± 4.9)	0.3^b^ (± 0.04)	83.7^b^ (± 13.9)	18.4^b^ (± 5.5)	18098^a^ (± 6657)	15.2^b^ (± 4.9)	23716^b^ (± 4406)	478^a^ (± 72.2)	24.5^b^ (± 4.9)
3	11.1^c^ (± 0.8)	20.4^b^ (± 1.7)	0.3^c^ (± 0.02)	70.7^b^ (± 5.2)	21.8^b^ (± 1.3)	20710^b^ (± 1477)	18.9^b^ (± 1.2)	25623^b^ (± 5773)	682^b^ (± 112)	32.7^c^ (± 3.6)
4	4.3^d^ (± 1.4)	10.7^c^ (± 2.5)	0.2^a^ (± 0.03)	43.5^a^ (± 15.5)	8.7^c^ (± 2.9)	7311^c^ (± 3166)	6.4^c^ (± 2.6)	11284^c^ (± 1897)	118^c^ (± 15.2)	13.1^a^ (± 3.8)
5	3.1^e^ (± 0.7)	7.6^c^ (± 1.8)	0.1^d^ (± 0.02)	27^a^ (± 8.5)	5.4^c^ (± 1.2)	4260^c^ (± 1142)	3.7^c^ (± 1.0)	10229c (± 3486)	174^c^ (± 122)	8.5^d^ (± 2.5)
*F value*	37*	18.5*	36.7*	30.9*	32.8*	23.3*	32.6*	21.1*	26.2*	48.2*

Means with different superscript letters within a column are significantly different. The mean and STD of the sediment metal concentrations based on spatial variation were calculated from metal concentrations recorded at each site during the eight sampling periods over the 2-year period

*Indicates the significant differences (*P* < 0.05)

The comparison of the sediment metal results with metal concentrations from elsewhere (South Africa, African continent, and international estuaries) is presented in Table 3. For all metals, except Zn, results from this study compare favorably with an earlier report from the Mhlathuze Estuary. Zinc concentrations were less than half of that recorded during the earlier study (Table 3). Mhlathuze Estuary metal concentrations from this study were all lower than in known contaminated areas in Richards Bay Harbour, but comparable or slightly higher than that from uncontaminated areas in the harbour. Results also show that Co, Pb, Cd, Ni, Cu, Fe, and Zn concentrations, when compared to estuaries outside Africa, were generally lower in the Mhlathuze Estuary. Comparison with sediment metals recorded in the St Louis Estuary in Senegal revealed that some metals such as Cd, Cu, and Zn were lower in the Mhlathuze Estuary, while others such as Co, Ni, and Mn were higher (Table 3).

**Table 3:** Comparison of the mean (± 1STD) sediment metal concentrations in the Mhlathuze Estuary and other estuaries from South Africa and from around the world: (1) present study, (2) Mzimela et al. 2014, (3) Wepener & Vermeulen, 2005, (4) Binning & Baird (2001), (5) Diop et al. (2015), (6) Duodu et al. (2017), (7) Hamzeh et al. (2014), (8) Banerjee et al. (2012), and (9) Wedepohl (1995)

Estuaries and coastal areas	Co (μg/g)	Pb (μg/g)	Cd (μg/g)	Cr (μg/g)	Ni (μg/g)	Al (μg/g)	Cu (μg/g)	Fe (μg/g)	Mn (μg/g)	Zn (μg/g)	Ref
South Africa											
Mhlathuze Estuary	6.4 ± 3.2	13.6 ± 5.1	0.2 ± 0.1	53.3 ±23.2	13.4 ± 6.7	12668 ± 6958	10.9 ± 6.2	17094 ± 7135	356 ± 230	18.8 ± 9.7	1
Mhlathuze Estuary	−	14.4 ± 3.3	−	66.3± 12.7	−	−	12.7 ± 2.3	21353 ± 9861	363 ± 76	46.9 ± 6.5	2
Richards Bay Harbour (cont)	−	−	−	152 ± 50.2	−	−	30.8 ± 15.3	42148 ± 9799	580 ± 80	129 ± 43.6	3
Richards Bay Harbour (uncont)	−	−	−	57.8 ± 16.9	−	−	−	18705 ± 6696	201 ± 81	58.3 ± 11.4	3
Swartkops Estuary	−	32.9 ± 27.6	−	20.3 ± 12.5	−	−	−	−	114 ±94	35.9 ± 26.6	4
International											
St Louis Estuary*, Senegal	3.6 ± 1.1	233 ± 35.5	0.68 ± 0.16	67.6 ± 18.1	7.9 ± 2.7	1814 ± 1710	46.5 ± 10.2	1819 ± 1783	55.7 ± 20.3	30.8 ± 7.1	5
Brisbane Estuary, Australia.	14.9 ± 2	25.6 ± 9.2	0.3 ± 0.06	15 ± 3	15.3 ± 2.3	−	−	15784 ± 2518	386 ± 428	106 ± 36	6
Seine Estuary, France	−	138 ± 6	6.2 ± 0.03	98 ± 5	31 ± 3	−	−	−	−	448 ± 20	7
Hooghly Estuary, India	18.2 ± 1.9	23.4 ± 1.9	2.0 ±0.1	40.1 ± 3.0	33.9 ± 6.8	−	−	2.86** ± 0.4	502 ± 51.8	53.4 ± 5.9	8
Average continental crust	11.6	17	0.10	35	18.6	77440	14.3	30890	527	52	9

*cont* contaminated areas, *uncont* uncontaminated area

*Indicates the value in mg/kg

**Indicates the value in %

### Relation between the trace metals in sediment and other physicochemical variables

**Fig. 3:**
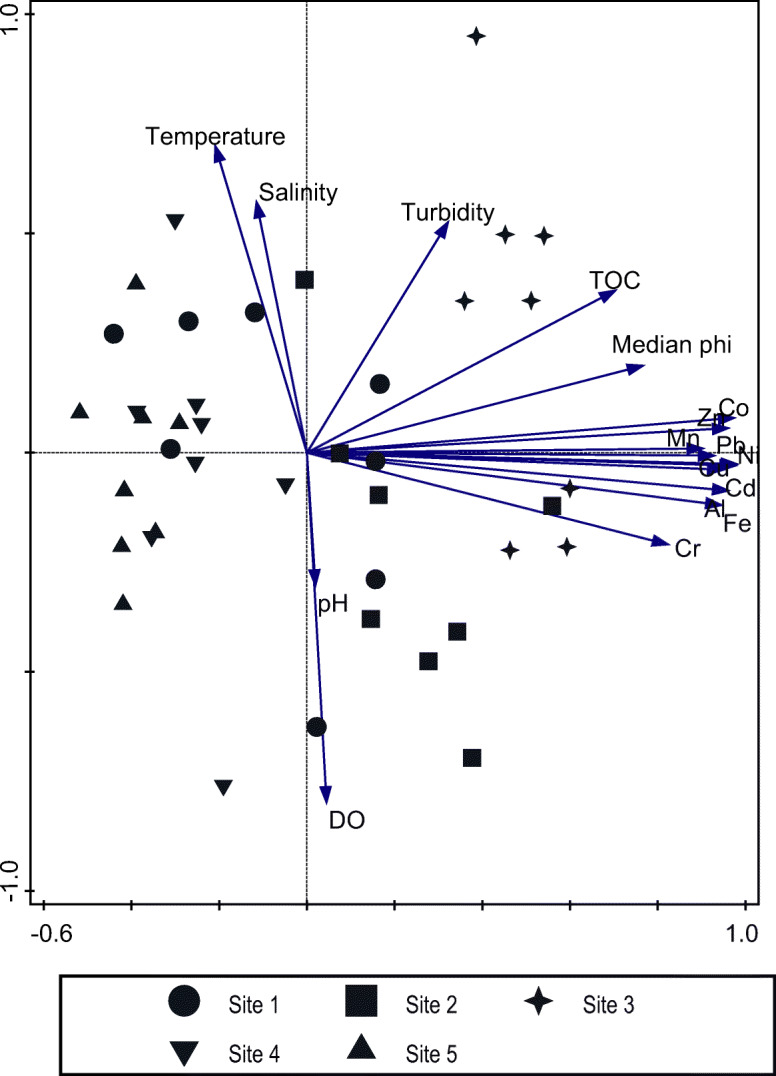
PCA biplot of the association between trace metals in sediments and environmental variables. The cumulative percentage for axes 1 and 2 was calculated as 71.4% (59.3% on the first and 12.1% on the second axis)

Principal component analysis (PCA) (Fig. 3) revealed strong positive correlations between Al and Fe concentrations and all other metals. Grain size and organic content showed strong positive correlations with all metals. In contrast, concentrations of all metals showed no or very weak correlations with salinity, temperature, pH, dissolved oxygen, and depth. Salinity was closely correlated with temperature, which is atypical for subtropical estuaries, suggesting higher salinities during summer. The highest concentrations of all metals were associated with sites 2 and 3, while lowest metal concentrations were associated with sites 1, 4, and 5. Eigenvalue for the PCA plot indicated that 71% of the variability was explained by the first two axes; thus, 29% of the variability was associated with variables other than that shown on the plot.

### Sediment pollution and toxicity assessment

Results for the three pollution indices for the five sites in the Mhlathuze Estuary are presented in Table 4. Enrichment factor (EF) results indicated no enrichment of Zn (EF < 1), minimal enrichment of Co, Pb, Cd, Ni, Cu, and Mn (EF 1-2), while Cr showed moderate enrichment (EF 2–5) at all sites with highest values at sites 1 and 2 (Table 4). The geoaccumulation index (Igeo) results indicated no contamination at all sites (Igeo < 0), with the exception of sites 2 and 3, where low Cr contamination (Igeo < 1) was recorded (Table 4). The pollution load index (PLI) results indicated no metal pollution (PLI < 1), except at site 3, where moderate pollution (PLI 1.12) was observed. The mean PLI value (PLI 0.68) confirms that the Mhlathuze Estuary is unpolluted (PLI < 1) (Table 4). There were significant differences in mean EF, PLI, and Igeo values between sampling sites (*P* < 0.05), but not between seasons (*P* > 0.05) (Table 5).

**Table 4:** Geoaccumulation index (Igeo), enrichment factor (EF), and pollution load index (PLI) results for sediment metals at five sites in the Mhlathuze Estuary

	Co		Pb		Cd		Cr		Ni		Cu		Mn		Zn		
Site	Igeo	EF	Igeo	EF	Igeo	EF	Igeo	EF	Igeo	EF	Igeo	EF	Igeo	EF	Igeo	EF	PLI
1	− 0.52	1.2	− 0.34	1.9	− 1.28	1.6	− 0.13	3.0	− 0.36	1.7	− 0.36	1.7	− 0.51	1.2	− 0.74	0.7	0.61
2	− 0.34	0.9	− 0.19	1.3	− 1.14	1.1		3.2	− 0.20	1.3	− 0.17	1.4	− 0.22	1.2	− 0.51	0.4	0.93
3	− 0.20	1.2	− 0.10	1.5	− 1.12	1.1		2.4	− 0.11	1.4	− 0.06	1.6	− 0.07	1.6	− 0.38	0.8	1.12*
4	− 0.62	1.1	− 0.39	1.8	− 1.38	1.4	− 0.11	3.4	− 0.52	1.3	− 0.55	1.3	− 0.83	0.6	− 0.79	0.7	0.44
5	− 0.76	0.9	− 0.53	1.5	− 1.56	1.1	− 0.31	2.4	− 0.72	0.9	− 0.78	0.8	− 0.73	0.9	− 0.98	0.5	0.31
mean	− 0.49	1.1	− 0.31	1.6	− 1.29	1.3	− 0.04	2.9	− 0.38	1.3	− 0.38	1.4	− 0.47	1.1	− 0.68	0.7	0.68

*Indicates the pollution exists, and values in red indicate unpolluted to moderately polluted

**Table 5:** One way analysis of variance (ANOVA) results for differences between mean pollution index values between sites and season in sediments of the Mhlathuze Estuary

Pollution index	ANOVA (*F* value)	
	Site	Season
Pollution load index (PLI)	48.5*	0.08
Enrichment factor (EF)	2.19*	0.54
Geoaccumulation index (Igeo)	37.70*	0.08

*Indicates a significant difference

**Fig. 4:**
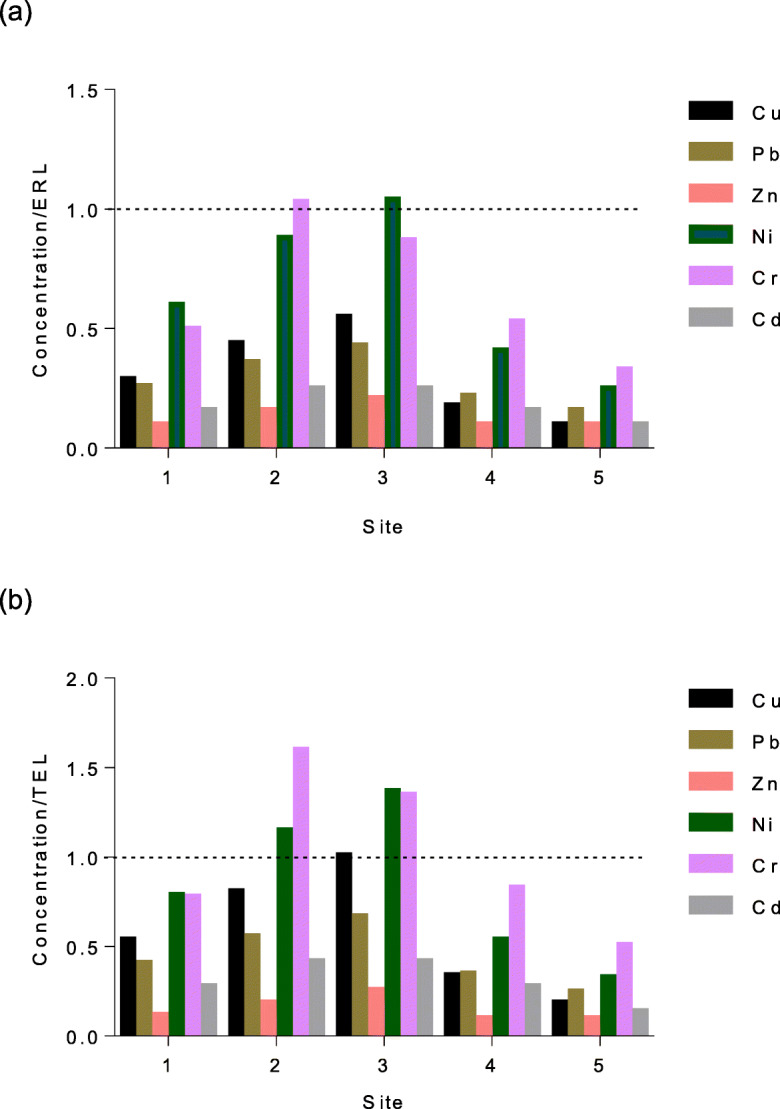
Comparison of trace metals in sediments of Mhlathuze Estuary with **a** ERL and **b** TEL guidelines

Sediment quality guidelines (SQGs) (ERL and TEL) were used to assess the toxicity of sediment metal concentrations obtained during this study (Fig. 4). Results indicate that toxic units (TC) for Cd, Pb, Cu, Ni, Cr, and Zn at sites 1, 4, and 5 were less than one using both ERL and TEL guidelines and is non-toxic to aquatic life. In contrast, Cr and Ni concentrations produced TC values of greater than one using TEL and ER guidelines, indicating potential toxicity.

### Characteristics of the macrobenthic fauna

A total of 87 macrobenthic taxa were recorded in the Mhlathuze Estuary (see Izegaegbe et al. 2020 for a detailed description of the macrobenthic community). Of the seven most abundant species, four were crustaceans, two were polychaetes, and one was an oligochaete (Table 6). These seven taxa accounted for 81% of the benthic organisms recorded. The tanaid *Halmyrapseudes cooperi* accounted for 51% of the benthic organisms recorded, followed by the oxypodid crab *Paratylodiplax blephariskios* (6%) and amphipod *Grandidierella bonnieroides* (5%). The mean number of taxa and species diversity is presented in Table 7. The number of taxa and species diversity was highest at sites 2 and 4, while lowest numbers were consistently recorded at the mouth of the estuary (site 5). There were significant differences in mean number of taxa (*F* = 6.0, *P* < 0.001) and species diversity (*F* = 5.9, *P* < 0.001) between sites, suggesting a strong spatial influence in the community structure. There were, however, no significant differences between seasons in the number of taxa (*F* = 0.3, *P* = 0.803) and species diversity (*F* = 0.3, *P* = 0.838).

**Table 6:** The faunal group, species, mean density per site, dominance, and cumulative dominance of the seven most abundant macrobenthic taxa in Mhlathuze Estuary

Faunal group	Species name	Mean density (N m^-2^)	Dominance (%)	Cum. dominance (%)
Crustacean	*Halmyrapseudes cooperi*	51771	50.74	50.74
Crustacean	*Paratylodiplax blephariskios*	6424	6.30	57.04
Crustacean	*Grandidierella bonnieroides*	5458	5.35	62.39
Polychaeta	*Prionospio sexoculata*	5280	5.17	67.56
Crustacean	*Americorophium triaeonyx*	4800	4.70	72.26
Polychaeta	*Mediomastus capensis*	4491	4.40	76.66
Oligochaeta	*Oligochaeta spp.*	3949	3.87	80.53

**Table 7:** Mean (± 1STD) number of macrobenthic taxa and species diversity at five sampling sites in Mhlathuze Estuary

Sites	Mean number of taxa	Mean species diversity
1	14 ± 5.5	2.5 ± 0.4
2	23.1 ± 8.9	3 ± 0.4
3	12.7 ± 5.1	2.3 ± 0.5
4	21 ± 9.4	2.9 ± 0.5
5	8.7 ± 3.4	2.1 ± 0.4

### Effect of sediment metals on macrobenthic community structure

**Fig. 5:**
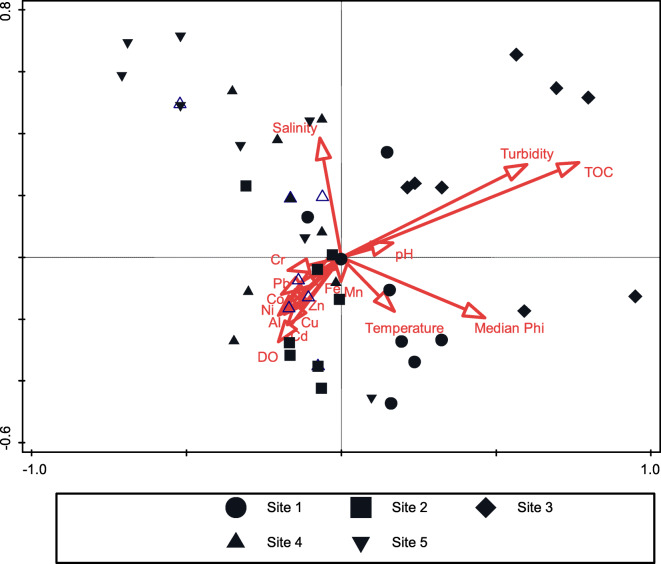
CCA triplot of the association between sediment metals in Mhlathuze Estuary, environmental variables, and macrobenthic assemblage

Canonical correspondence analysis (CCA) was used to measure the correlation between environmental variables, sediment metal concentrations, and the macrobenthic community (Fig. 5). The two axes shown (axis 1: 35.2%, axis 2: 27.3%) explained 62.5% of the variability. The CCA plot indicated that variability in the macrobenthic community was largely driven by a combination of environmental variables such as grain size, TOC, turbidity, and salinity, with minor influence from sediment metal concentrations. Of all variables included in the analysis, TOC (pseudo *F* = 2.9, *P* = 0.002) was the most important in structuring the macrobenthic community, followed by turbidity (pseudo *F* = 2.1, *P* = 0.002) and grain size (pseudo *F* = 2.0, *P* = 0.002). All three variables showed a significant correlation with the community structure. None of the metals showed a strong correlation with the macrobenthic community. The analysis also indicated that high Cr, Pb, Co, Zn, Cu, Al, Ni, and Cd concentrations were correlated with the communities at sites 2 and 4, and not with the site 3 community as expected. The plot also showed a strong correlation between TOC and turbidity and the macrobenthic community at site 3, suggesting a strong influence of sediment characteristics on the site 3 community. The effect of the relatively low sediment metal concentrations on the assemblage was therefore completely overshadowed by natural environmental variables related to granulometry (sediment grain size, TOC, and turbidity) and river flows (salinity).

### Assessment of M-AMBI index and correlation between other indices

**Fig. 6:**
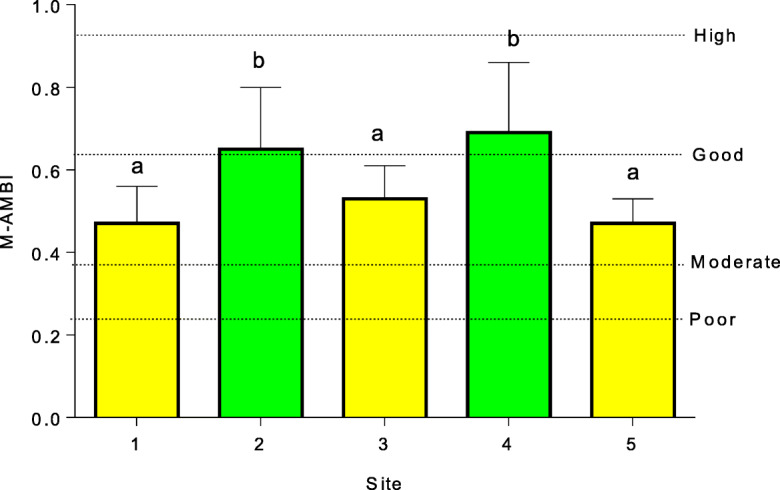
Macrobenthic habitat quality (mean (± STD) M-AMBI, *n* = 40) at the five sites in the Mhlathuze Estuary. Bars with common superscript do not differ significantly (The mean and STD of macrobenthic habitat quality based on spatial variation were calculated from M-AMBI scores derived for each site during the eight sampling periods over the 2-year period)

M-AMBI results (Fig. 6) revealed that macrobenthic habitat quality was significantly higher at sites 2 and 4 (Fig. 6), with these areas being categorized as good habitat quality. Sites 1, 3, and 5 were categorized as a moderate habitat quality. There were no significant seasonal differences in mean M-AMBI scores (*P* > 0.05). M-AMBI showed a weak negative correlation with enrichment factor (EF) and pollution load index (PLI), suggesting that metal concentrations at the five sites had little influence on macrobenthic habitat quality. In contrast, a significant positive correlation was observed between PLI and EF (Table 8).

**Table 8:** Pearson correlation between M-AMBI, enrichment factor (EF), and pollution load index (PLI) in the Mhlathuze Estuary during 2016–2017

Indices	M-AMBI	EF MEAN
M-AMBI	–	
EF Mean	− 0.09	–
PLI-8	− 0.14	0.97**

*Correlation is significant at the 0.01 level (two-tailed)

**Correlation is significant at the 0.05 level (one-tailed

## Discussion

The study revealed that sediment metal concentrations in the Mhlathuze Estuary were consistently higher in subtidal mudflats sediment (site 3) and lowest at the mouth of the estuary (site 5), with significant differences between sites in all metals, largely due to inter-site variation in sediment characteristics. Metal concentrations in the estuary were generally low, as measured against the sediment quality guidelines (SQGs), with only Ni and Cr showing potential toxicity to aquatic life. The PLI confirmed that the estuary is relatively unpolluted, with only site 3 being moderately polluted. Significantly higher numbers of taxa and species diversity were recorded at sites 2 and 4. There was a strong correlation between the macrobenthic community and environmental variables, notably grain size, TOC, and turbidity, but not between the macrobenthic community and sediment metal concentrations, suggesting that metals had a limited influence on the community structure. The biotic index M-AMBI showed the macrobenthic habitat at sites 2 and 4 to be of good quality, while sites 1, 3, and 5 were moderate habitat quality.

The salinities measured during the study reflected the strong marine influence in the estuary. The relatively high mean salinity in the upper reaches (site 1) is atypical for a subtropical estuary with a large catchment, reflecting the negative influence of extensive water abstraction from the river on the natural flow regime of the estuary. Site 3, in the middle of the embayment, was typically muddy and had the highest TOC levels. Coarse-grained sediments with a low TOC was typically found in the upper reaches (site 1) and at the mouth of the estuary (site 5).

A progressive increase was seen in concentrations of sediment metals from the upper reaches (site 1) to the mangrove-fringed lower end of the Mhlathuze River (site 2) and through to the shallow subtidal mudflat (site 3), followed by a decline in concentrations in the channel habitat (site 4) through to the marine dominated sandy substrate at the mouth (site 5). The high sediment metal concentrations recorded in mudflat sediment (site 3) can be attributed to the high mud and TOC content, while the lack of strong tidal currents in this area results in a net import and deposition of sediment-bound metals onto the mudflat. High loads of trace metals are often associated with consolidated mud on subtidal mudflats (Magesh et al. 2011), largely due to the fine-grained sediment and high organic content (Orr et al. 2008). The mouth of the estuary (site 5) showed consistently lowest sediment metal concentrations, which can be attributed to the strong tidal currents typical of a permanently open estuary mouth, which results in coarser sediment and low TOC. Strong tidal currents typically prevent deposition of fine sediments, resulting in relatively low sediment metal accumulation in the marine dominated sandy substrate (Acevedo-Figueroa et al. 2006).

Compared to earlier sediment metal records for the Mhlathuze Estuary (1997–1998) (Mzimela et al. 2014), mean concentrations of Pb, Cr, Al, Cu, Fe, Mn, and Zn during the present study were consistently lower, suggesting a decline in sediment metal concentrations over the past 20 years (Table 3). This was observed at all sites with comparable data. The mean Zn concentration, in particular, was much lower during the present study (46.8 μg/g vs 18.8 μg/g). On the mudflat (site 3), where highest metal concentrations were recorded, concentrations of Cr, Cu, and Zn were 34%, 17%, and 56% lower during the present study, respectively. Cyrus et al. (2008) reported that earlier dredging for expansion of the coal terminal in the adjacent Richards Bay Harbour leads to significant sediment intrusion into the Mhlathuze Estuary, resulting in increased turbidities and fine sediment deposition. Dredging in the harbour could therefore have caused the higher metal concentrations reported during 1997–1998.

Compared to known contaminated muddy sediments in Richards Bay Harbour (Wepener & Vermeulen 2005) (Table 3), mean concentrations of Cr, Cu, and Zn recorded in muddy sediments (site 3) (Table 2) during this study were 53%, 40%, and 75% lower, respectively. This attests to the difference in the extent of industrial influence between the two systems. Compared to known uncontaminated sediments in Richards Bay Harbour (Wepener & Vermeulen 2005), mean Cr and Cu concentrations in muddy sediments (site 3) (Table 2) during this study were slightly higher, while Zn was 44% lower (Table 3). Concentrations of Pb and Zn in muddy sediments from the Mhlathuze Estuary were 38% and 9% lower than in the Swartkops Estuary in the Eastern Cape, respectively, whereas Cr and Cu concentrations were much higher (Binning & Baird 2001).

Concentrations of most metals in the sediment during this study were relatively low compared to concentrations reported in large, permanently open estuaries elsewhere (Table 3). Concentrations of Co, Pb, and Cu in muddy sediments (site 3) during the present study were 16%, 20%, and 60% lower than that reported for Brisbane Harbour sediments, respectively, while Zn and Cr concentrations were 6% and 370% higher (McCready et al. 2006). Compared to the highly industrialized Seine Estuary in France, concentrations of Pb, Cu, and Zn in muddy sediments (site 3) during the present study were 85%, 86%, and 93% lower, respectively, while the Cr concentration was only 28% lower (Hamzeh et al. 2014). As in the Brisbane Estuary (15 μg/g), mean Cr concentration in the Hooghly Estuary (40.1 μg/g) (Banerjee et al. 2012), which serves one of the biggest industrial and shipping areas of India, was much lower than in muddy sediments in the Mhlathuze Estuary (70.7 μg/g). These results suggest high Cr concentrations in the relatively unindustrialized Mhlathuze Estuary against global standards and are the cause for concern. The generally low metal concentrations during this study is probably be due to the highly regulated flows in the Mhlathuze River coupled with the lack of direct industrial influence in the estuary, with only indirect industrial effluent exposure from the adjacent port and the catchment. Other than a temporary dune-mining operation and a sugar mill along the regulated lower reaches of the Mhlathuze River during the study period, there are no other major industrial operations in the lower catchment.

The strong positive correlation between sediment metal concentrations and particle size and TOC was not unexpected and highlights the profound natural effect of granulometry and organic content on metal concentrations in estuarine sediments (Rainbow 2002; Akan et al. 2013). According to Kumar et al. (2017), the correlation between sediment texture and metal concentrations provides a basis in south-east India for determining the controlling factor that influences metal mobility. The strong correlation between sediment metal concentrations and sediment characteristics were clearly evident in the PCA plot (Fig. 3), with all metals showing a strong positive correlation with grain size, TOC, and turbidity. This confirms that concentrations of these metals were largely influenced by natural granulometry processes. The influence of turbidity is best attributed to the resuspension of muddy sediments due to the shallow nature of the estuary. This has implications for the bioavailability of metals to estuarine macrobenthic fauna (Shirneshan et al. 2012). The spatial distribution and relatively high concentrations of Cr suggest that its input may have been influenced by factors other than sediment characteristics, although the source of the high levels of Cr at sites 2 and 3 is not clear and requires further investigation.

The results of the three pollution indices used in this study confirmed their relevance as reliable biomonitoring tools for sediment pollution assessment in estuaries. Enrichment factor (EF) has been widely applied as a means to distinguish between metals arising from natural and anthropogenic contributions. The moderate enrichment of Cr observed at all sites may possibly be arising from the adjacent harbour as significant enrichment of Cr was recorded at the nearby bulk terminal and coal terminal in Richards Bay Harbour (Izegaegbe 2020). The EF values for Co, Pb, Cd, Ni, Cu, Mn, and Zn during this study reflected natural background levels, suggesting negligible enrichment. The geoaccumulation index (Igeo) values for Cr at sites 2 and 3 were indicative of sediments approaching moderate contamination. In contrast, Igeo values for Co, Pb, Cd, Ni, Cu, Mn, and Zn were indicative of uncontaminated sediments. The pollution load index (PLI) value for the muddy embayment (site 3) suggested moderate pollution in this area, in contrast to all other sites, which were categorized as unpolluted. The mean PLI value suggested that the Mhlathuze Estuary was generally unpolluted.

Sediment quality guidelines (SQGs) play an important role in pollution assessment by highlighting potential toxicity of sediment metals to aquatic biota (Zhang et al. 2017). Concentrations of Cu, Pb, Zn, Ni, Cr, and Cd were compared to available effect range low (ERL) and threshold effect level (TEL) SQG values. The toxic unit values (calculated as the observed metal concentration relative to the corresponding ERL/TEL concentration) for Cd, Cu, Pb, and Zn were generally less than 1, indicating that concentrations of these metals in sediments were within the recommended range and that adverse effects will rarely occur (Muchaa et al. 2003). Nickel and Cr concentrations at sites 2 and 3 were indicative of potential toxicity to aquatic fauna, based on TEL values. Chromium at site 2 and Ni at 3 slightly exceeded the ERL values, indicating that observed Cr and Ni concentrations are potentially toxic to aquatic life in the Mhlathuze Estuary.

Correspondence analysis of the correlation between the macrobenthic community structure and environmental variables and sediment metal concentrations clearly showed that variation in the community structure was largely influenced by natural variation in sediment characteristics (grain size, TOC, and turbidity) and not by sediment metal concentrations. There was a significant positive correlation between the community structure and grain size, TOC, and turbidity. In contrast, a weak correlation was observed between community structure and sediment metals. In the metal-polluted Fal Estuary, Warwick (2001) reported that sediment metal concentrations correlated strongly with the macrobenthic community. Similarly, a significant correlation was reported between macrobenthic community structure and trace metals in the polluted Paraguacu estuarine system in Brazil (Barros et al. 2008). In the present study, sediment metal concentrations are best correlated with the macrobenthic community at site 2. Results, however, indicate that macrobenthic diversities remained high at sites 2 and 4 (Izegaegbe et al. 2020), suggesting that metal concentrations at these sites did not adversely affect macrobenthic diversity. The unpolluted status of site 2, as evident in the PLI analysis, lends further support to the fact that sediment metal concentrations did not affect the macrobenthic community. The Mhlathuze Estuary is relatively isolated from direct industrial influences with no significant enrichment of metals at sites 2 and 4. The macrobenthic assemblage on the mudflat (site 3) was strongly influenced by granulometry (muddy substrate, high TOC, and high turbidity) rather than sediment metals, even though highest metal concentrations were recorded in this area. Macrobenthic organisms are typically strongly influenced by the nature of the sediment in which they live (Vivier 2010). In the mildly polluted Hawkesbury Estuary, south-eastern Australia, the macrobenthic community structure was similarly strongly correlated with environmental variables such as TOC, grain size, and salinity rather than sediment metals (Macfarlane & Booth 2001).

The M-AMBI scores showed that the macrobenthic habitat quality at sites 2 and 4 was good, with these areas supporting diverse macrobenthic communities with many sensitive macrobenthic taxa. Sites 1, 3, and 5 were categorized as macrobenthic habitat of moderate quality. Site 3 was associated with relatively high sediment metal concentrations, notably Cr and Ni, as evident in the high PLI values. It is therefore not surprising that the macrobenthic community in this area reflected lower habitat quality. In contrast to site 3, sites 1 and 5 were relatively unpolluted with low PLI scores and were therefore not exposed to high sediment metal concentrations, yet the habitat quality status was rated as moderate rather than good. The reason for this may be related to natural sediment granulometry, as the substrate at site 1 was fine sand and influenced by freshwater input, while site 5 was typically coarser marine sand. The nature of the sediments in these areas probably do not support as diverse macrobenthic communities compared to the organically rich, muddy mangrove habitat (site 2).

The negative correlation between M-AMBI and pollution indices (EF and PLI) confirmed the suitability of M-AMBI as a bioindicator of functional macrobenthic habitat quality in estuarine habitat quality assessments (Bigot et al. 2008; Borja & Tunberg 2011; Sigamani et al. 2015). In the pollution stressed Nandgaon Estuary, India, AMBI and M-AMBI were found adequate in assessing macrobenthic habitat quality status by differentiating between sites exposed to differing levels of pollution (Sivaraj et al. 2014). It is however a concern that in the present study, the macrobenthic habitat quality of the moderately polluted mudflat (site 3) was rated similar to that of the unpolluted sandy mouth area (5). This reveals the difficulty with using M-AMBI in low to mildly stressed systems, such as the Mhlathuze Estuary. In such systems, interpretation of the M-AMBI results should be done with caution, as the index cannot differentiate between sites with naturally low species diversity caused by environmental variables such as sediment particle size and sites with depressed species diversity due to anthropogenic stressors such as metal pollution. As such, in these systems, it is best to use AMBI and M-AMBI results in combination for assessment of habitat quality, as AMBI relies purely on the sensitivity of taxa to pollution and anthropogenically induced habitat disturbance, without the confounding influence of naturally low species diversity. The continued use of these indices as a biomonitoring tool to assess the habitat quality of the Mhlathuze Estuary is, however, highly recommended, given the high national conservation importance of the estuary. The proposed expansion of the adjacent Richards Bay Harbour to cater to the increasing national and regional industrial development is anticipated to increase the anthropogenic pressure on receiving estuarine ecosystems. In conclusion, the low sediment metal concentrations observed during this study, in combination with the relatively high macrobenthic habitat quality scores, suggest that habitat quality in the Mhlathuze Estuary is still relatively good and that the system is worthy of its high national conservation status (Turpie et al. 2002). All efforts must be made to ensure that the conservation status of the estuary is maintained, particularly in view of the proposed future expansion of the adjacent port.

The development and implementation of sediment quality guidelines for South African estuarine and coastal waters to reflect local conditions are strongly recommended. Finally, findings from this study could serve as a baseline for future sediment toxicity research and ecological quality assessments in subtropical permanently open estuaries, especially as this is the first report on the use of biotic indices such as M-AMBI to assess sediment metal contamination and associated macrobenthic habitat quality in Southern Africa.

## Electronic supplementary material

ESM 1 (DOCX 31 kb)
