# Supplementary material for: Trace metal contamination in sediment in the Mhlathuze Estuary, northern KwaZulu-Natal, South Africa: effects on the macrobenthic community
Source: Environ Monit Assess. 2020 May 29;192(6):401. doi: 10.1007/s10661-020-08352-9 (PMC7256079; doi:10.1007/s10661-020-08352-9)
Supplement: Supplementary file 1 — (DOCX 31 kb) [file 10661_2020_8352_MOESM1_ESM.docx]

**Appendix A** Species name and M-AMBI assignation number

| Species name | Assignation number |  |
| --- | --- | --- |
| *Amphipod sp1* | (i) |  |
| *Amphipod sp2* | (i) |  |
| *Amphipod sp3* | (i) |  |
| *Ancistrocylis parva* | (iii) |  |
| *Aonides oxycephala* | (iii) |  |
| *Halmyrapseudes cooperi* | (ii) |  |
| *Armandia intermedia* | (i) |  |
| *Capitella capitata* | (v) |  |
| *Capitellidae sp1* | (v) |  |
| *Capitellidae sp2* | (V) |  |
| *Ceratonereis keiskamma* | (II) |  |
| *Corophidae sp* | (iii) |  |
| *Monocorophium acherusticum* | (iii) |  |
| *Americorophium triaenonyx* | (iii) |  |
| *Cossura coasta* | (iv) |  |
| *Dendronereis arborifera* | (iv) |  |
| *Desdemona ornata* | (ii) |  |
| *Dosinia hepatica* | (i) |  |
| *Eriopsa chilkensis* | (i) |  |
| *Euchone capensis* | (ii) |  |
| *Eumarcia paupercula* | (ii) |  |
| *Fabricia sp* | (ii) |  |
| *Gastropod sp1* | (i) |  |
| *gastropod sp2* | (i) |  |
| *Glycera convoluta* | (ii) |  |
| *Glycera longipinus* | (ii) |  |
| *Glycera natalensis* | (ii) |  |
| *Glycera papillosa* | (ii) |  |
| *Glycera subaena* | (ii) |  |
| *Glycera unicornis* | (ii) |  |
| *Grandidierella bonnieroides* | (i) |  |
| *Heteromastus filiformis* | (iii) |  |
| *Hirudinea sp* | (iv) |  |
| *Hymenosoma orbiculare* | (i) |  |
| *Iphinoe truncata* | (i) |  |
| *Leptochelia barnardi* | (iii) |  |
| *Lumbrinereis cavifrons* | (ii) |  |
| *Lumbrinereis latreilli* | (ii) |  |
| *Lysidice sp* | (ii) |  |
| *Macoma littoralis* | (ii) |  |
| *Magelona cincta* | (i) |  |
| *Magelona sp* | (i) |  |
| *Marphysa sanguinea* | (ii) |  |
| *Marphysa sp* | (ii) |  |
| *Mediomastus capensis* | (iv) |  |
| *Megalomma sp* | (ii) |  |
| *Mesopodopsis africana* | (ii) |  |
| *Nainneris laevigata* | (i) |  |
| *Natica sp* | (ii) |  |
| *Naussarius kraussianus* | (II) |  |
| *Nematonereis unicornis* | (ii) |  |
| *Nemerteans sp* | (iii) |  |
| *Nephtys capensis* | (ii) |  |
| *Nephtys dibranchis* | (i) |  |
| *Nephtys hombergi* | (ii) |  |
| *Nephtys spearocirrata* | (ii) |  |
| *Nereis sp* | (iii) |  |
| *Notomastus latericeus* | (iii) |  |
| *Notomastus sp* | (iii) |  |
| *Oligochaetes* | (v) |  |
| *Owenia fusiformis* | (ii) |  |
| *Paratylodiplax blephariskios* | (i) |  |
| *Phyllodoce malmgreni* | (ii) |  |
| *Pilargis falcata* | (ii) |  |
| *Platyhelminthes sp* | (ii) |  |
| *Poldora normalis* | (iv) |  |
| *Polychaete sp* | (iv) |  |
| *Polydora kempi* | (iii) |  |
| *Polydora sp* | (iv) |  |
| *Prionopsio pinnata* | (iv) |  |
| *Prionospio cirrifera* | (iv) |  |
| *Prionospio saldana* | (iv) |  |
| *Prionospio sexoculata* | (iv) |  |
| *Pygospio elegans* | (iii) |  |
| *Sabellid sp* | (i) |  |
| *Scolepsis squamata* | (iii) |  |
| *Scoloplos uniramus* | (iii) |  |
| *Scoloplos marsupialis* | (i) |  |
| *Sipunculid sp* | (i) |  |
| *Solen cylindraceus* | (ii) |  |
| *Spionidae spp* | (iii) |  |
| *Syllidae sp1* | (ii) |  |
| *Syllidae sp2* | (ii) |  |
| *Tellina capsiode* | (i) |  |
| *Tharyx filibranchia* | (iv) |  |
| *Tharyx marioni* | (iv) |  |
| *Urothoe serrulidartylus* | (i) |  |

**Appendix B** Mean (STD) values of AMBI, diversity, species richness and M-AMBI scores of macrobenthic community in Mhlathuze Estuary with ANOVA and post hoc test inserted below

| Mean | | | | | STD | | | |
| --- | --- | --- | --- | --- | --- | --- | --- | --- |
| Site | AMBI | Diversity | Richness | M-AMBI | AMBI | Diversity | Richness | M-AMBI |
| 1 | 3.54 | 2.24 | 14 | 0.47 | 1.17 | 0.79 | 5.55 | 0.09 |
| 2 | 2.2 | 2.45 | 23.12 | 0.65 | 0.45 | 1.02 | 8.93 | 0.15 |
| 3 | 1.29 | 1.63 | 12.75 | 0.53 | 0.27 | 0.46 | 5.12 | 0.08 |
| 4 | 2.36 | 3.24 | 21 | 0.69 | 0.41 | 0.96 | 9.38 | 0.17 |
| 5 | 2.81 | 2.41 | 8.62 | 0.47 | 0.85 | 0.44 | 3.54 | 0.06 |

| ANOVA | | | | | |
| --- | --- | --- | --- | --- | --- |
|  | | | | | |
|  | Sum of Squares | df | Mean Square | F | Sig. |
| Between Groups | .345 | 4 | .086 | 6.216 | .001 |
| Within Groups | .486 | 35 | .014 |  |  |
| Total | .831 | 39 |  |  |  |

Post Hoc Tests

| Multiple Comparisons | | | | | | |
| --- | --- | --- | --- | --- | --- | --- |
| Dependent Variable: MAMBI | | | | | | |
| LSD | | | | | | |
| (I) sites | (J) sites | Mean Difference (I-J) | Std. Error | Sig. | 95% Confidence Interval | |
|  |  |  |  |  | Lower Bound | Upper Bound |
| site 1 | site 2 | -.18516^*^ | .05892 | .003 | -.3048 | -.0656 |
|  | site 3 | -.06221 | .05892 | .298 | -.1818 | .0574 |
|  | site 4 | -.22214^*^ | .05892 | .001 | -.3417 | -.1025 |
|  | site 5 | -.00137 | .05892 | .982 | -.1210 | .1182 |
| site 2 | site 1 | .18516^*^ | .05892 | .003 | .0656 | .3048 |
|  | site 3 | .12295^*^ | .05892 | .044 | .0033 | .2426 |
|  | site 4 | -.03697 | .05892 | .534 | -.1566 | .0826 |
|  | site 5 | .18380^*^ | .05892 | .004 | .0642 | .3034 |
| site 3 | site 1 | .06221 | .05892 | .298 | -.0574 | .1818 |
|  | site 2 | -.12295^*^ | .05892 | .044 | -.2426 | -.0033 |
|  | site 4 | -.15992^*^ | .05892 | .010 | -.2795 | -.0403 |
|  | site 5 | .06084 | .05892 | .309 | -.0588 | .1805 |
| site 4 | site 1 | .22214^*^ | .05892 | .001 | .1025 | .3417 |
|  | site 2 | .03697 | .05892 | .534 | -.0826 | .1566 |
|  | site 3 | .15992^*^ | .05892 | .010 | .0403 | .2795 |
|  | site 5 | .22077^*^ | .05892 | .001 | .1012 | .3404 |
| site 5 | site 1 | .00137 | .05892 | .982 | -.1182 | .1210 |
|  | site 2 | -.18380^*^ | .05892 | .004 | -.3034 | -.0642 |
|  | site 3 | -.06084 | .05892 | .309 | -.1805 | .0588 |
|  | site 4 | -.22077^*^ | .05892 | .001 | -.3404 | -.1012 |

| *. The mean difference is significant at the 0.05 level. |
| --- |
